# Enhancing nursing student presentation competences using Facilitatory Pecha kucha presentation pedagogy: a quasi-experimental study protocol in Tanzania

**DOI:** 10.1186/s12909-023-04628-z

**Published:** 2023-09-03

**Authors:** Setberth Jonas Haramba, Walter C. Millanzi, Saada A. Seif

**Affiliations:** Department of Nursing Management and Education, The University of Dodoma, Dodoma, Tanzania

**Keywords:** Learning satisfaction, Nursing students, Pecha Kucha presentation pedagogy, Presentation knowledge, Presentation skills

## Abstract

**Introduction:**

With an increasing number of nursing students in higher education institutions in Tanzania, traditional student presentation pedagogies are insufficient to enhance effective learning. Pecha Kucha presentation is a new promising approach that can improve students’ speaking skills, learning process, creativity, and students’ engagement in learning. It involves the use of 20 slides where each covers 20 s, thus making a total of 6 min and 40 s. The current study will assess the effect of Pecha Kucha’s presentation on presentation knowledge, skills, and learning satisfaction among nursing students in Tanzania.

**Objectives:**

The objectives of this study aimed to determine the baseline and end-line of Pecha Kucha Presentation knowledge, skills, and learning satisfaction among nursing students.

**Methods:**

This study protocol proposes to employ an uncontrolled quasi-experimental study design with a quantitative approach among 230 university nursing students in Dodoma Region using simple and stratified sampling techniques. It proposes to employ the researcher-administered questionnaire to assess study variables that arise as students use the Pecha Kucha presentation format to prepare and present their assignments. The study will involve training of research assistants, pre-assessment of study variables, and training, and demonstration of Pecha Kucha presentations format among study participants. It will also involve assigning topics to study participants, submission and evaluation of the prepared assignments, participants’ presentations in the classroom, post-intervention assessment, data analysis, reporting and dissemination of the study findings.

**Conclusion:**

This study will address and complement the global need to invest in nursing in an attempt to prepare competent nurses who are capable of solving complex health challenges through critical thinking, analysis, collaboration, and effective communication. The study will inform policymakers, health training institutions, and educators about a new engaging, and innovative nursing student presentation approach that enhances students’ creativity, critical thinking skills, and meaningful learning. The referred nursing students’ presentation approach intends to equip the students with survival and life skills in the 21st century in an attempt to meet the global economy and job opportunities.

**Trial registration:**

It is not applicable as this is not a trial.

## Introduction

Nurses are front-line healthcare professionals in health promotion, disease prevention, treatment, and rehabilitation and they constitute about 19.3 million health professionals globally [1–3]. They are heroes playing a critical role in the health care system, emergency management, and response [1]. Africa has about 12.89 per 10, 000 population displaying a great shortage, and needs to increase efforts in preparing competent graduate nurses with the varieties of skills to increase the nursing and midwifery workforce, thereby meeting sustainable development goals 2030 and universal health coverage [4]. To increase the nursing workforce, nursing education needs to be reoriented on preparing competent nurses and midwives who will drive primary healthcare services to meet society’s needs [2, 3].

Nursing students are needed to have different skills acquired during learning to enable them to provide nursing care and management to society [5]. The referred nursing care and management practices include identifying, analyzing, synthesizing, and solving health-related problems, and communicating effectively within and between healthcare professionals [5]., Given an increasingly global economy and international competition for jobs and opportunities, the current traditional classroom learning methods are insufficient to meet such 21st -century challenges and demands [6].

The integration of problems solving, presentation, creativity, innovation, collaboration, information, and media literacy skills helps to overcome the noted challenges among students [6–8]. The skills in question constitute the survival skills that help the students not only for carrier development and success but also for the personal, social and public quality of life as they enable students to overcome 21st challenges upon graduation [6].

To enhance nursing students’ participation in learning, stimulating their critical thinking, self-learning, life skills, and satisfaction with the learning process, a combination of teaching and learning pedagogy needs to be employed [9, 10]. Among others, lectures, classroom presentations, group discussions, problem-based learning, demonstrations, reflection, and role-playing are commonly used for those purposes [9].

Health training institutions in Tanzania shall strive to prepare competent and skilled health professionals to solve primary and tertiary healthcare challenges [11]. The training institutions are also required to prepare skilled and competent nurses who shall ensure, promote and protect public health and safety [12–14]. Furthermore, only graduate nurses who demonstrate skills and required competencies are eligible to be registered and are allowed to provide nursing and services [12].

Ineffective and non-interactive learning which contributes to poor critical thinking, problem-solving, and decision-making skills has been reported by several scholars. Poor use and design of student PowerPoint presentations have led to confusing graphics due to many texts in the slides and reading of about 80 slides [15–17]. Indeed, this non-interactive learning causes boredom and tiresome among the learners which is usually evidenced by glazing eyes, long yawning, occasional snoring, use of phone, and frequent trips to the bathroom [15, 17].

With an increasing number of nursing students in higher education institutions, traditional student presentation pedagogies are insufficient to engage students in the teaching and learning process and stimulate their effective learning presentation skills, collaboration teamwork skills, innovation, creative, and meaningful learning in an attempt to solve health challenges [18, 19]. Indeed, insufficiency of the traditional student presentation pedagogies leads to limited opportunities for students’ classroom presentations, thereby limiting their creativity, innovation, presentation skills, collaboration, and team works skills.

Pecha Kucha’s presentation is a new promising approach for students learning in the classroom context as it motivates learners’ self-directed and collaborative learning, learner creativity, innovations, presentation skills, collaboration, and students’ engagement in learning [20–22]. Pecha Kucha’s presentation encourages students to read more materials online and library and cooperative learning among learners and is interesting and enjoyable among students [23].

Pecha Kucha presentation originated from the Japanese word “*chit chat”* which represents the fast-paced presentation used in different fields, including teaching, marketing, advertising, and designing [24–26]. It involves 20 slides where each slide is shown in 20 s, thus making a total of 6 min and 40 s for the whole presentation [22].

For effective learning through Pecha Kucha presentation, the design, and format of the presentation should be meaningfully limited to 20 slides and targeted to 20 s for each slide and should be rich in content of the presented topic using high-quality images or pictures attuned to content knowledge, message to be delivered medium and target audiences [22, 24]. Each slide should contain a primordial message with a well-balanced amount of information. In other words, the message should be simple in the sense that each slide should contain only one concept or idea with neither too much nor too little information, thus making it easy to be grasped by the audience [22, 25, 27].

The “true spirit” of Pecha Kucha is that it mostly consists of powerful images and meaningful specific text. Rather than the presenter reading text from the slides, an image and short phrases should communicate the core idea while the speaker offers well-rehearsed and elaborated comments. The presenter should master the subject matter and incorporate the necessary information from classwork [22, 28].

It enhances the students’ critical thinking about the preparation of information and image to be presented to an audience, thus enhancing self-engagement in learning and practicing the flow of content to make it meaningful. Pecha Kucha’s presentation helps the students to be focused on the key content to be presented, create effective, simple, and clear visual points, and rehearse effectively before going to the audience [29]. The same study revealed that Pecha Kucha’s presentation guarantees good time management, makes the student confident and it engages the audiences during the presentation [29].

The audience’s engagement in learning by paying attention and actively listening to Pecha Kucha’s presentation was higher than in traditional PowerPoint presentations [30]. The creativity and collaboration during designing, selecting the appropriate images and contents, rehearsal before the presentation, and discussion after each presentation made students satisfied by enjoying Pecha Kucha presentation compared to traditional presentations [30, 31]. The time management and students’ self-regulation were found significant through Pecha Kucha presentation among students and the teachers or instructors could plan appropriately the times for classroom instruction [31, 32].

However, little is known about Pecha Kucha’s presentation in nursing education in Sub-Saharan African countries, Tanzania in the list, as there are insufficient evidence regarding the research(s) that have been published on the description of its meaning and effect on enhancing students’ presentation competencies. Little is also known about Pecha Kucha presentations among nurses globally. Thus, this study will assess the effect of Pecha Kucha’s presentation pedagogy in enhancing presentation skills among nursing students. In particular, the study will largely focus on the knowledge of Pecha presentation format, skills, and learning satisfaction during the preparation and presentation of the student’s assignment project work, case reports, or field reports.

The study will answer the null hypothesis; H_0_ = H_1_: which hypothesizes that there is no significant difference in nursing student’s classroom Pecha Kucha presentation knowledge, skills, and learning satisfaction scores between baseline and end-line assessments. The association between Pecha Kucha’s presentation and student knowledge, skills, and learning satisfaction will be formulated and analyzed before intervention and after the intervention.

The study will form the basis for developing new presentation pedagogy among nursing students to stimulate effective learning and acquisition of 21st -century skills in the era of the increased competitive knowledge-based society, changing of nature, and technological eruption. It will also form the basis to redefine classroom practices in an attempt to enhance and transform nursing students’ learning experiences. To achieve this, the study will determine the baseline and end-line nursing student’s Pecha Kucha presentation knowledge, nursing student’s skills during the preparation of classroom presentations, and nursing student’s learning satisfaction with classroom presentations in Tanzania.

## Methods and materials

### Study area

This study will be conducted in health training institutions in Tanzania. Tanzania has a total of 47 registered public and private universities and university colleges that offer health programs ranging from certificate to PhD levels [33, 34]. A total of 7 out of 47 universities offer a bachelor of science in nursing, 4 universities offer a master’s program in nursing and midwifery sciences, and a PhD [33].

To enhance the representation of nursing students in Tanzania, this study will be conducted in Dodoma Municipal Council which is one of Tanzania’s 30 administrative regions [35]. Dodoma Region has two [2] universities that offer nursing programs at diploma and degree levels [36]. The referred universities host a large number of nursing students compared to the other five [5] universities in Tanzania with traditional students’ presentation approach predominating nursing students’ teaching and learning process [33, 37].

The two universities under study include the University of Dodoma and St. John’s University of Tanzania, which are located in Dodoma Urban District. The University of Dodoma is a public university hosting about 28,225 undergraduate students, 724 postgraduate students, and 142 training programs [35]. St. Johns University is a non-profit private legally connected with the Christian-Anglican Church [38]. It has a student enrollment ranging from 5000 to 5999 and it provides courses and programs leading to higher education degrees in a variety of fields, including pre-bachelor degrees, bachelor degrees, and master’s degrees [39]. It has 766 nursing students pursuing a bachelor of science in Nursing and 113 nursing students pursuing a diploma in Nursing in the academic year 2022/2023 [38, 39].

### Study design and approach

**Fig. 1:**
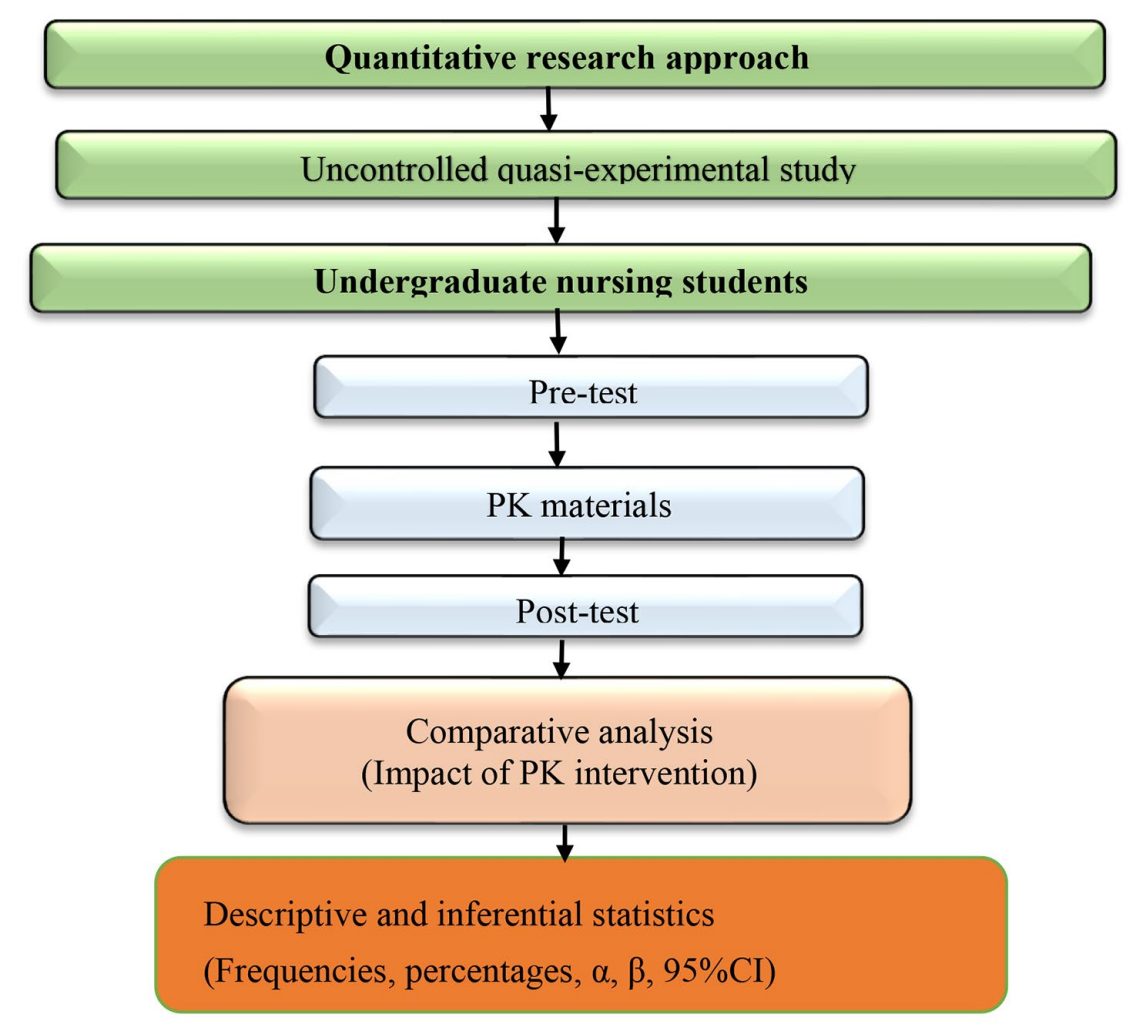
A flow diagram of the study design and approach [40]

An uncontrolled quasi-experimental design with a quantitative research approach will be used to establish the quantifiable data on the participants’ socio-demographic characteristic profiles and outcome variables under study. The design will involve pre and post-tests to determine the effects of the intervention on the aforementioned outcome variables. The design will involve three phases baseline data collection process (pre-test via a cross-sectional survey), implementation of the intervention (process), and end-line assessment (post-test) as shown in Fig. 1. All methods will be carried out under both international and national research ethics and institutional postgraduate guidelines.

### Target population

The study proposes to involve nursing students pursuing a Diploma in Nursing and a Bachelor of Science in Nursing in Tanzania.

### Study population

The study proposes to involve first-year to third-year nursing students pursuing a Diploma in Nursing and first-year to fourth-year nursing students pursuing a Bachelor of Science in Nursing.

- The population is highly expected to demonstrate the competencies and mastery of different survival and life skills to work independently at various levels of health facilities within and outside Tanzania.
- The cohort of undergraduate nursing students will involve adult learners who can set goals, develop strategies to achieve their goals, and, hence positive professional behavioral outcomes.
- Currently, there is a large number of enrolled undergraduate nursing students, thus making it an ideal population for intervention and will approximately serve as a good representation of the universities offering nursing program [19, 37]. Moreover, as per annual data, the average number of graduate nurses ranges from 3500 to 4000 from all colleges and universities in the country [41].

### Inclusion criteria

The study proposes to include nursing students pursuing a Diploma in Nursing and a Bachelor of Science in Nursing. The referred students should be registered at the university during the time of study and live on or off campus. They should also not be exposed to PK training and should have regular classroom attendance. This will enhance the enrollment of adequate study samples from each study program and monitoring of study intervention and easy control of confounders.

### Exclusion criteria

The study participants with visual, language, hearing, and self-reported sicknesses will be excluded from the study. This is because the study adopted the Multimedia Theory of Cognitive Learning (MTCL) which involves effective learning through audio-visual, hence such participants may neither effectively benefit from the intervention nor effectively participate in developing and presenting their assignments, project works, case reports or field reports through Pecha Kucha presentation.

Also, the study may fail to hire special personnel to demonstrate and interpret language for them. Furthermore, those participants who will be reported sick during the study may fail to comply with the requirement of the study, which needs them to prepare and present their assignment, project works, case reports, or field reports through Pecha Kucha presentation.

### Sample size determination

An uncontrolled quasi-experimental study on Pecha Kucha as an alternative to traditional PowerPoint presentations, at Worcester University, United States of America, reported a significantly high student engagement during Pecha Kucha presentation compared to traditional PowerPoint presentations [30]. The mean score for the classroom with a traditional PowerPoint presentation was 2.63 while the mean score for the Pecha Kucha presentation was 4.08. This study proposes to adopt the formula which was used to calculate the required sample size for an uncontrolled quasi-experimental study [42]:

$${\rm{n = }}\frac{{{{\{ {Z_\alpha }\surd {\rm{[\pi o(1 - \pi o)] + }}{{\rm{Z}}_{\rm{\beta }}}\surd {\rm{[\pi 1(1 - \pi 1)]\} }}}^{\rm{2}}}}}{{{{{\rm{(\pi 1 - \pi o)}}}^{\rm{2}}}}}$$

Where: Zα was set at 1.96 from the normal distribution table.

Zβ was set at 0.80 power of the study.

Mean zero (π0) is the mean score of students’ engagement in using PowerPoint presentation = 2.63.

Mean one (π1) is the mean score of student engagement in using the Pecha Kucha presentation = 4.08.

$$\begin{array}{l}{\rm{n}}\,{\rm{ = }}\,\frac{{{{\{ 1.96\surd {\rm{[2}}{\rm{.63(1}}\,{\rm{ - }}\,{\rm{2}}{\rm{.63)]}}\,{\rm{ + }}\,{\rm{0}}{\rm{.80}}\surd {\rm{[4}}{\rm{.08(1}}\,{\rm{ - }}\,{\rm{4}}{\rm{.08)]\} }}}^{\rm{2}}}}}{{{{{\rm{(4}}{\rm{.08}}\,{\rm{ - }}\,{\rm{2}}{\rm{.63)}}}^{\rm{2}}}}}\\{\rm{n}}\,{\rm{ = }}\,\frac{{{{{\rm{\{ - 8}}{\rm{.40 + - 12}}{\rm{.566\} }}}^{\rm{2}}}}}{{{{{\rm{(1}}{\rm{.45)}}}^{\rm{2}}}}}\\{\rm{n}}\,{\rm{ = }}\,\frac{{{\rm{439}}{\rm{.57}}}}{{{\rm{2}}{\rm{.105}}}}\\{\rm{n}}\,{\rm{ = }}\,{\rm{209}}\,{\rm{ + }}\,{\rm{10\% }}\,{\rm{non - response}}\,{\rm{rate}}\end{array}$$

The total sample size will comprise **230** nursing students.

### Sampling technique

Given the availability of higher training institutions that offer undergraduate nursing programs, the study proposes to use a simple random sampling technique to obtain the universities in which the current study will be conducted. Based on the target population, the study proposes to use the purposeful sampling technique to select the colleges or schools of nursing from the selected universities. It also proposes to use a convenience sampling technique to obtain classes of undergraduate nursing students pursuing a Diploma in Nursing and Midwifery and a Bachelor of Science in Nursing from the study area, which will be available at the time of the study.

To establish representation for a minimum sample from each class and by gender, the proportionate stratified sampling technique (sample size/population size× stratum size) will be used as recommended by scholars [43]. The study also proposes to use the simple random sampling technique to obtain the required sample size from each strata where after obtaining lists of each strata, cards with code numbers will be allocated for each class and strata, mixed with empty cards depending on the strata size.

Both cards will be put into different pots, and labeled appropriately by their class and strata names. Upon arriving in the specific classroom and after introduction, the research assistant will ask each nursing student to draw one card from the respective strata pot. Those who will select cards with code numbers will be recruited in the study with their code numbers as the participation identity numbers. The process will continue for each class until the required sample size will be obtained.

To ensure the effective participation of nursing study in the study, the research assistant will work hand to hand with facilitators, lecturers of the respective classrooms, the head of the department, and class leaders. The importance, advantages, and disadvantages of participating in the study will be given to the study participant during the recruitment process to create awareness and remove possible fears. Also, during the intervention study participants will be given pens, and notebooks to enable participants to take notes and bites will be provided during training sessions. Furthermore, the attrition rate has been considered for the non-response rate during recruitment and intervention to ensure the required sample is obtained.

**Fig. 2:**
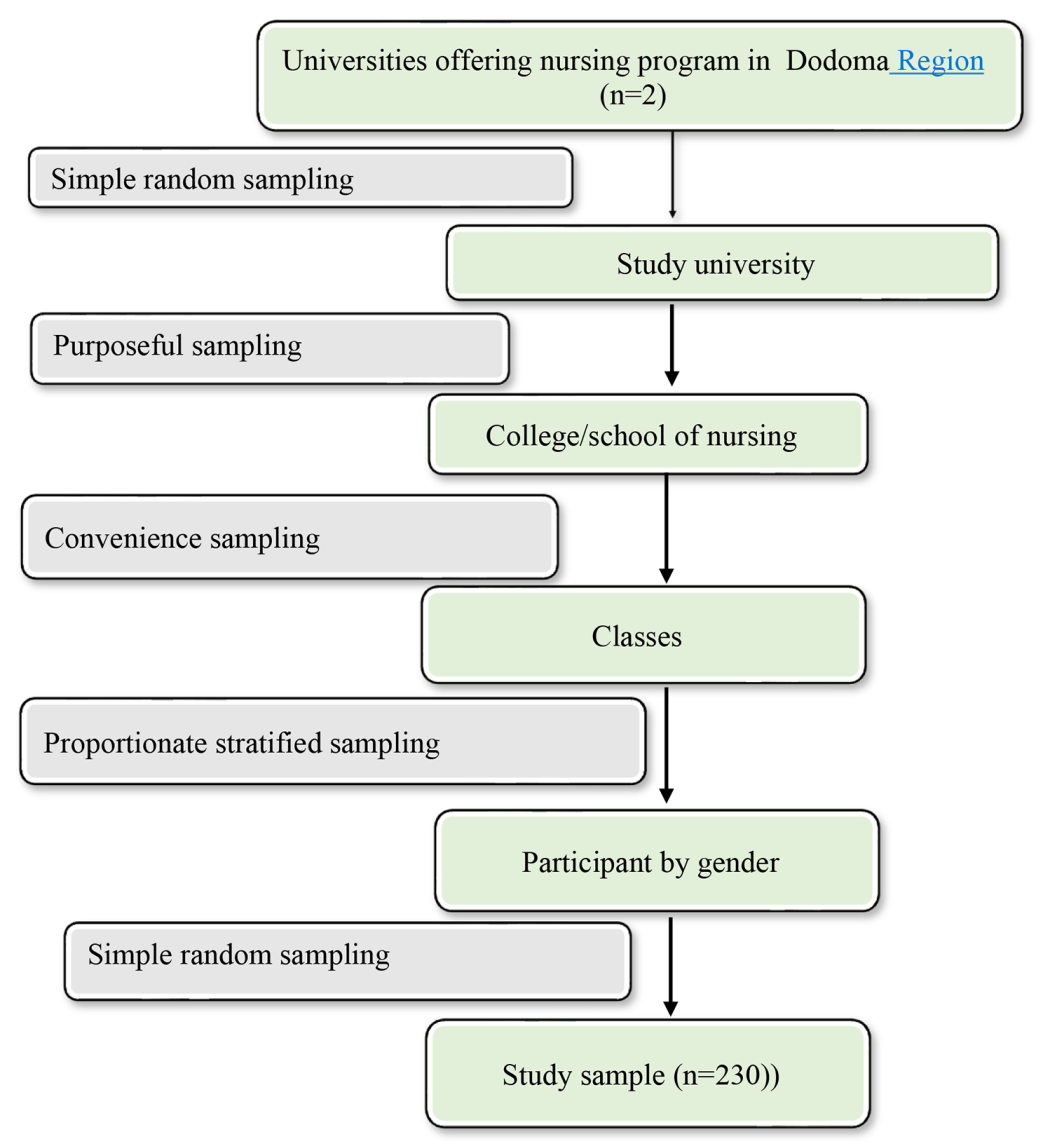
A flow diagram of the proposed sampling procedures (Study plan, 2023)

The proposed study sampling procedures being illustrated in Fig. 2:

### Data collection method

The study proposes to use the researcher-administered questionnaire to collect the participants’ socio-demographic information, co-related factors, Pecha Kucha presentation knowledge, preparation, and presentation skills. It will also gather the students’ learning satisfaction views as they use the Pecha Kucha presentation format to prepare and present their assignments, project works, and case or field reports. This will ensure the clarity and well participants’ understanding of all question items before providing the appropriate responses.

The data will be collected by research assistants in the classroom with the study participants sitting at a distance to ensure privacy, confidentiality, and quality of information that will be provided by the research participants. The research assistant will guide and lead the study participant to answer and fill in the information on the questionnaire for each section, domain, and question item. The research assistant will also collect the baseline information (pre-test) before the intervention, which will be compared with the post-intervention information. This will be done in the first week of the data collection period after the training of the research assistant and recruitment of study participants.

Using the researcher-administered questionnaire, the research assistant will also collect the participants’ information related to the knowledge of Pecha Kucha presentation, skills, and learning satisfaction as it is used to prepare and present their project work and case or field reports after the intervention. The study also proposes to review the prepared presentation using the Pecha Kucha rubric to assess the organization, visual appeal and creativity, content knowledge, and adherence to Pecha Kucha presentation requirements. The referred review will be conducted during the intervention where each study participant will be assigned to prepare and submit the slides before the presentation. Furthermore, the participant’s ability to share and communicate the given assignment will be observed in the classroom presentation using Pecha Kucha materials.

### Data collection tools

The study will adapt and modify the students’ Pecha Kucha knowledge from the official Pecha Kucha website, and students’ questionnaire on presentation skills that were used by scholars [26–28, 44]. Also, the study will adapt and modify students’ questionnaires on learning satisfaction as used by scholars [45–47]. The modification will consider the language used, complexity, and context of the tools used to ensure clarity, literacy level, and cultural norms of participants.

The data collection tool will consist of four sections whereby each section will try to explore distinct information. Section one will consist of 68 question items that will assess the socio-demographic characteristics of the study participants. Section two will consist of 49 question items that will assess the student’s knowledge of the Pecha Kucha presentation with a particular focus on its definition, origin, characteristics, advantages, disadvantages, and how to develop it.

Section three [3] will consist of 33 question items that will assess the student’s skills during the preparation and presentation of the assignment, project work, case reports, or field reports using the Pecha Kucha presentation format. The referred assessment will specifically focus on the student’s ability to prepare the presentation content, master the learning content, share presentation materials, and communicate their understanding to audiences in the classroom context.

Section four [4] will consist of 29 question items which will assess the student’s learning satisfaction through overall learning experience during the preparation and presentation of the student’s assignments, project works, case or field reports, and learning autonomy and audience’s engagement during the presentations. All items in sections three and four will be rated on a five-point Likert scale which ranges from 5 = strongly agree, 4 = agree, 3 = not sure, 2 = disagree to 1 = strongly disagree while section two will be measured using “Yes/No” and “I do not know responses”.

The Pecha Kucha rubric which was adopted from the official Pecha Kucha website will also be used to assess the student’s ability to develop and present their assignments [22]. The rubric will assess the student’s ability to develop a 20 × 20 presentation and organize the presentation, images and ideas, quality, and appropriate text/images chosen. Moreover, it will assess creativity and content knowledge in terms of topic coverage and examples. The items will be rated on a ten-point Likert scale which ranges from 10 = Exemplary (Pecha Rocked It) 8 = Accomplished (Pecha You Betcha), 6 = Developing (Pecha Next Time) and 4 = Beginning (Pecha Train Wreck).

### Validity and reliability of research instruments

Validity of the research instrument refers to whether the measuring research instrument measures the behaviors or quality that is intended to be measured and is a measure of how well the measuring instrument performs its function [48]. There are four forms of validity, namely face validity, content, criteria, and construct. The structured questionnaire which intends to assess the participants’ knowledge and skills and learning satisfaction will be validated for face validity and content validity.

The principal investigator will initially develop and modify the question items for different domains of student learning literature on the Pecha Kucha presentation and its implication on student learning. The items will be shared and discussed with two [2] educationists, two [2] research experts, one [1] statistician, and supervisors to ensure clarity, appropriateness, adequacy, and coverage of the student’s knowledge, skills, and learning satisfaction.

The content validity test will be used until the saturation of the expert opinions and inputs is achieved. The inter-observer rating scale on a five-point Likert scale ranging from 5-points = very relevant to 1-point = Not relevant will also be used. The sharing and discussion process will involve addition, input deletion, correction, and editing of the relevance, appropriateness of the content for the study participants, and scope of the content. The consulted research experts’ and supervisor’s comment sections will also involve correcting grammatical issues in the instruments and rewriting different domains in an attempt to improve clarity based on the literacy level of study participants.

Their observations will also be considered and the revised tools will be revised and shared for the finalization before the actual data collection task. Irrelevant items will be modified or deleted based on the consensus that will be reached with the consulted experts and supervisors before subjecting the questionnaires to the pre-test.

Reliability of the research instruments refers to the ability of the research instruments or tools to provide similar and consistent results when applied at different times and circumstances [48]. This study will adapt the tools and question items used by different scholars to assess the impact of PKP on student learning. To ensure the reliability of the tools, the pilot study will be conducted in one of the nursing training institutions to assess the complexity, readability, clarity, completeness, length, and duration of the tool.

Ambiguous and difficult (left unanswered) items will be modified or deleted based on the consensus that will be reached with the consulted experts and supervisor before subjecting the questionnaires to the pre-test. The study proposes to include 10% of undergraduate nursing students from an independent geographical location for a pilot study. The findings from the pilot study will be subjected to explanatory factor analysis (Set a ≥ 0.3) and scale analysis to determine internal consistency of the tools using the Cronbach alpha of ≥ 0.7 which will be considered reliable [49–51].

### Variables definitions

#### Pecha Kucha presentation

This is a specific style of presentation whereby the presenter delivers the content using 20 slides which are dominated by images or pictures, tables, or figures. Each slide is displayed for 20 s, thus making a total of 400 s (6 min and 40 s) for the whole presentation.

#### Pecha Kucha presentation knowledge

This involves the students’ understanding of the meaning, origin, characteristics, advantages, and disadvantages of the Pecha Kucha rubric.

#### Skills in this study

This involves the students’ ability to plan, prepare, master learning content, create presentation materials and share them with peers or audience in the classroom. They constitute the learning activities that stimulate creativity, innovation, critical thinking, and problems solving skills.

#### Student learning satisfaction

It involves short-term attitude and perception which result from the evaluation of the student’s learning experiences during the preparation and presentation of their assignments through Pecha Kucha presentation.

### Measurement of students’ knowledge of pecha kucha presentation

The student’s knowledge of the meaning of Pecha Kucha presentation and its origin, characteristics, advantages, and disadvantages, and how to develop Pecha Kucha materials and presentation will be measured using 49 question items. The study participants will ask to supply “Yes” or “No” responses to demonstrate their understanding. The participants who will correctly score the highest marks above the mean (mean = 24.5) from the total of 49 points will be considered more knowledgeable while those who will score less than the mean score will be considered less knowledgeable.

### Measurement of pecha kucha preparation and presentation skills

The student’s skills will be measured using the four [4] learning domains. The first domain constitutes the students’ ability to plan and prepare presentation content. It consists of 17 question items that assess the student’s ability to gather select information, search specific content to be presented in the classroom, find learning content from different resources, and search literature materials for the preparation of the assignment using Pecha Kucha format. The referred assignment will also aim to ascertain a deeper understanding of the contents or topic, learning ownership, and motivation to learn topics with a clear understanding and ability to identify the relevant audience, and segregate and remove unnecessary content using the Pecha Kucha format.

The second domain constitutes the student’s mastery of learning during the preparation and presentation of their assignment to the audience in the classroom. It consists of six [6] question items that measure the student’s ability to re-read and rehearse before the classroom presentation and practice harder the assignment and presentation. It also measures the student’s ability to evaluate the selected information and content before their actual presentation and make revisions for the selected information and content before the presentation using the Pecha Kucha format.

The third domain constitutes the students’ ability to prepare the presentation materials. It consists of six [6] question items that measure the student’s ability to organize the information and contents, prepare the classroom presentation, revise and edit presentation resources, materials, and contents and think about the audience and classroom design. The fourth domain constitutes the students’ ability to share their learning. It consists of four [4] question items that measure the student’s ability to communicate their learning with the audience, present new understanding to the audience, transfer the learning to the audience and answer the questions about the topic or assignment given.

The variable will be measured using a 5-point Likert scale, thus making a total of 165 scores. The high skills in preparing and presenting assignments will be measured using the highest score above the mean (M = 82.5 points) from a total of 165 points. A low score below the mean will be considered as low skills in preparing and presenting assignments using the Pecha Kucha presentation format among study participants.

### Measurement of learning satisfaction through pecha kucha presentation

Students learning satisfaction will be measured using three satisfaction domains adopted from the antecedent learning satisfaction scale. The first domain constitutes the learning experience. It consists of 16 question items that assess if the participants enjoy preparing and presenting their assignment, the need to prepare more and/or practice harder to master the topic, the difficulty of the presentation format, and a better understanding of the topic or assignment. The referred items also assess the usefulness of the assignments/learning resources provided for learning, student engagement with the topic, motivation, and interest to learn more about the topic, and responsibility to learn. The first domain will be measured using a 5-point Likert scale, thus making a total of 80 scores.

The second domain constitutes the participants’ learning autonomy. It consists of eight [8] question items that aim to assess if the participants enjoy finding information on the given topic, taking responsibility for learning, owning the topic or assignments, presenting and answering the questions, and student’s ability to choose the content and resources for presentation. The second domain will be measured using a 5-point Likert scale, thus making a total of 40 scores.

The third domain constitutes the audience’s engagement during the presentation. It consists of five [5] question items that assess the audience’s focus during presentations, the ability to draw the audience’s attention and interest by preventing them from charting with friends during the presentation, the ability of the presentations to make the audience note important information conveyed and ability of the audience to listen actively and attentively. The third domain will be measured using a 5-point Likert scale, thus making a total of 25 scores.

High learning satisfaction will be measured by determining the highest scores above the mean (72.5 points) from the total of 145 points while low learning satisfaction will be measured using low scores below the mean.

### Implementation of the intervention

The implementation of the study will involve the training of research assistants, sampling of the study participants, setting of the venue, pre-assessment of the student’s knowledge, skills, and learning satisfaction, training and demonstration of Pecha Kucha presentations to study participants, assigning the topics to study participants and preparation of the assignment. The implementation of the study will also involve the participants’ submission of their assignments to the PI (Principal Investigator) for evaluation, the participants’ presentation of their assigned topic using Pecha Kucha format, and the post-intervention assessment of the student’s knowledge, skills, and learning satisfaction, data analysis and reporting.

The intervention will involve the PI and two [2] trained research assistants. The intervention in question will base on the concept of Multimedia Theory of Cognitive Learning (MTCL) for enhancing effective learning in the 21st century.

### Training of research assistants

Two research assistants will be trained concerning principles, characteristics, and format of the Pecha Kucha presentation using the curriculum from the official website and oriented to the rubric that will be used to assess students’ assignments. The research assistant will also be oriented to the data collection tools and methods of data collection in an attempt to ensure the relevancy and appropriate collection of the participants’ information.

### Schedule and duration of training among research assistants

The PI will prepare the training schedule based on the negotiation and consensus with the research assistants. Moreover, the PI will train the research assistants to assess learning and how to collect the data using the questionnaire and maintain the privacy and confidentiality of the study participants.

### Descriptions of the intervention

As shown in Table 1, the intervention will be conducted among the nursing students at the University of Dodoma which are found in Dodoma Region, Tanzania Mainland after obtaining their consent. The participants will be trained in the principles and characteristics of Pecha Kucha presentation and how to prepare and present their assignments or projects or field reports using the Pecha Kucha presentation format. The training will be accompanied by one example of an ideal Pecha Kucha presentation on the topic which will be selected by the PI.

After the training session, the evaluation will be conducted to assess the participants’ understanding of the Pecha Kucha conceptualization, its characteristics, and its principles. Each participant will be given a topic as an assignment based on the fundamentals of nursing, medical nursing, surgical nursing, community health nursing, mental health nursing, emergency critical care, pediatric, reproductive and child health, midwifery, communicable diseases, non-communicable diseases, orthopedic and cross-cutting issues in nursing as recommended by scholars [30, 52].

The study participants will be given 14 days for the preparation, rehearsal, and presentation using the Pecha Kucha presentation format and submission of prepared slides to the research assistant and PI for evaluation before the actual classroom presentation. The evaluation of the participants’ assignments will involve the number of slides, quality of images used, number of words, organization of content and messages to be delivered, slide transition, duration of presentation, flow, and organization of slides. Afterward, each participant will be given 6 min and 40 s for presentation and 5 to 10 min for answering the questions on the topic presented as raised by other participants. Based on the number of study participants, an average of 4 participants will be allowed to present their assignments every hour.

After completion of the presentations, the research assistant will assess the participant’s knowledge and skills which will result in meaningful and satisfactory learning using the researcher-administered questionnaire. The collected data will be analyzed in an attempt to ascertain the association between the Pecha Kucha presentation and outcome variables of interest compared with the baseline information. The intervention session will be conducted in selected classrooms which will accommodate all participants at the time that will be arranged by the class coordinators and institution administrators. The summary of the intervention is described in Table 1.

**Table 1:** Descriptions of the interventions

Aspects of the intervention	Descriptions
Sample size	230
Baseline Assessment	Pre-test
Intervention materials	PK guidelines and curriculum for the school, Pecha Kucha presentations, Pecha Kucha videos, projectors, learning pictures/images, notebook and pens, flip charts, marker pens, computers, learning notes
Topics	Pecha Kucha Presentation
Sessions	• Concepts of Pecha Kucha presentation • Principles and characteristics of Pecha Kucha presentation • Advantages and disadvantages of Pecha Kucha presentation • How to develop Pecha Kucha presentation
Timing	1 0.5 session
Duration	3 h
Venue	Classrooms
Facilitators	Principle Investigator and research assistant
Mode of delivery	Lecture, discussion, and demonstration
Learners’ roles	Listening, taking some notes, asking questions
Facilitators’ roles	Training how to design and develop Pecha Kucha presentations Demonstrating Pecha Kucha’s presentation
Assignments	Preparation of individual Pecha Kucha presentation Submission and presentation of the assignment in classroom context using Pecha Kucha presentation format
Class presentations	Presentation in the entire class of study participants, answering questions, defending and addressing queries
Between presentation evaluation	Pecha Kucha presentation rubric
Session feedback	Session Evaluation
End-line assessment	Post-test (immediate after intervention)

**Source**: Study plan (2023)

### Evaluation of the intervention

The research assistant will be exposed to baseline information on students’ learning through the usual classroom presentation before Pecha Kucha training. The questionnaire containing question items on knowledge, skills, and learning satisfaction among the study participants will be used. After every presentation, there will be 5 to 10 min for classroom discussion and reflection on the content presented, which will be guided by the research assistant. During this time, the participants will get an opportunity to ask questions, get clarification from the presenter and give their opinion on how the instructional messages were presented, content coverage, area of the strength and weaknesses for improvement, and academic growth.

After completion of the presentation sessions, the research assistant will provide the questionnaire to participants to determine their knowledge about the Pecha Kucha presentation and learning experience, presentation skills during the preparation of their assignments, and classroom presentations. Furthermore, the research assistant will use the Pecha Kucha presentation rubric to observe the presenters’ adherence to the Pecha Kucha presentation framework and guidelines and levels of mastery.

### Data analysis

The findings from this study will be analyzed using the Statistical Package for Social Science (SPSS) computer software program version 26 for each variable and objective. Each outcome variable will be analyzed where the percentages, frequencies, frequency distributions, means, and standard deviations will be calculated and the results will be presented using figures, tables, and graphs.

The descriptive statistical analysis will be used to analyze the demographic information for the participants in an attempt to determine the frequencies and percentages of their distributions. A mean score analysis will be used to measure participants’ Pecha Kucha presentation knowledge, presentation skills, and learning satisfaction. A paired sample t-test will be used to compare the mean score differences of the outcome variables at baseline and end-line assessments. The mean scores differences will be determined based on the baseline scores against the post-intervention scores to establish any change in terms of knowledge, skills, and learning satisfaction among the study participants.

The factors associated with and development of participants’ Pecha Kucha presentation knowledge, presentation skills, and learning satisfaction will be established using linear regression analysis set at a 95% confidence interval and a 5% (≤ 0.05) significance level to accept or reject the null hypothesis. A detailed description of the data analysis for each objective and variable is indicated in Table 2.

**Table 2:** Data analysis plan

Objective	Variables	Method of measurement
To determine the effect of Pecha Kucha presentation pedagogy on nursing students’ presentation knowledge in Tanzania	**Independent variable**: Pecha Kucha presentation pedagogy, socio-demographic information, and co-related factors **Dependent/outcome variables**: Pecha Kucha’s presentation knowledge	• The objective will be measured on an ordinal scale • The students’ knowledge will be measured using 49 multiple multiple-choice question items. • The mean score analysis will be computed and the participant who will score equal to or above the mean (mean ≥ 24.5 scores) from the total of 49 scores will be considered knowledgeable and the participant who will score less than the mean score will be considered less knowledgeable. • Descriptive statistical analysis will be used to analyze participants’ demographic information to determine the frequencies and percentages of their distributions. • Paired sample t-test will be used to compare the mean score differences of Pecha Kucha presentation knowledge between baseline and end line • The linear regression analysis model will be used to assess factors associated with participants‘ Pecha Kucha presentation knowledge at baseline and at endline
To determine the effect of Pecha Kucha presentation pedagogy on nursing student’s skills during the preparation of classroom presentations in Tanzania	**Independent variable**: Pecha Kucha presentation pedagogy, socio-demographic information, and co-related factors **Independent/outcome variables**: Presentation Preparation and presentation skills	• The objective will be measured on an ordinal scale. • The participants’ presentation preparation skills will be measured using 33 question items using a 5-point Likert scale, thus making ma total of 165 points. • The descriptive statistical analysis will be used to analyze the participants’ demographic information in an attempt to determine the frequencies and percentages of their distributions. • The mean score analysis will be computed whereby the participant with the highest score above the mean (M ≥ 82.5 points) from the total of 165 points will be considered as high skilled whereas the low score below the means will be considered as low presentation skills. • The paired sample t-test will be used to compare the mean score differences of presentation skills between baseline and end-line assessment • The linear regression analysis model will be used to assess factors associated with participants’ presentation skills at baseline and at endline
To determine the effects of facilitatory Pecha Kucha presentation pedagogy on nursing students’ learning satisfaction in Tanzania	**Independent variable**: Facilitatory Pecha Kucha presentation pedagogy, socio-demographic information, and co-related factors **Independent/outcome variables**: Learning Satisfaction	• The objective will be measured on an ordinal scale. • The participants’ learning satisfaction will be measured using 29 question items on a 5-point Likert scale, thus making a total of 145 points. • The descriptive statistical analysis will be used to analyze the participant’s demographic information in an attempt to determine the frequencies and percentages of their distributions. • The mean score analysis will be computed whereby the highest score above the mean (M ≥ 72.5 points) from the total of 145 points will be considered as high learning satisfaction whereas the low score below means will be considered low learning satisfaction. The paired sample t-test will be used to compare the mean score differences of learning satisfaction between baseline and end-line assessment • The linear regression analysis will be used to measure factors associated with participants’ learning satisfaction at baseline and at endline

## Discussion

The findings of this study will be used to compare and complement the existing evidence in an attempt to unveil the knowledge, practices, skills, and effective learning through the Pecha Kucha presentation format. This will provide the answers to the purpose and research hypothesis through the association between the Pecha Kucha presentation and the outcome variables which will be drawn before and after the intervention. The methodology which will be employed during the study will be synthesized, analyzed, and compared with other scholars’ works on Pecha Kucha Presentation. The study limitations and strengths will also be thoroughly narrated in an attempt to inform future studies and researchers.

## Data Availability

The datasets that will be generated and analyzed by this study will be available from the corresponding author on reasonable request through harambasetberth@gmail.com & setberth.haramba@tnmc.go.tz.

## Abbreviations

Dr.: Doctor (PhD)
MTCL: Multimedia Theory of Cognitive Learning
NACTVET: National Council for Technical and Vocational Education and Training
PI: Principle Investigator
PK: Pecha Kucha presentation
SPSS: Statistical Package for Social Sciences
TCU: Tanzania Commission for Universities
WHO: World Health Organization
